# *Trifolium pratense* L. as a Potential Natural Antioxidant

**DOI:** 10.3390/molecules19010713

**Published:** 2014-01-07

**Authors:** Sanja Vlaisavljevic, Biljana Kaurinovic, Mira Popovic, Maja Djurendic-Brenesel, Bojana Vasiljevic, Dragoljub Cvetkovic, Sanja Vasiljevic

**Affiliations:** 1 Department of Chemistry, Biochemistry and Environmental Protection, Faculty of Sciences, Trg Dositeja Obradovica 3, Novi Sad 21000, Serbia; E-Mails: sanja.vlaisavljevic@dh.uns.ac.rs (S.V.); mira.popovic@dh.uns.ac.rs (M.P.); bojana.vasiljevic@dh.uns.ac.rs (B.V.); 2 Clinical Center of Vojvodina, Department for Forensic Medicine, Hajduk Veljkova 7-9, Novi Sad 21000, Serbia; E-Mail: maja.brenesel@gmail.com; 3 Faculty of Technology, University of Novi Sad, Bulevar Cara Lazara 1, Novi Sad 21000, Serbia; E-Mail: cveled@uns.ac.rs; 4 Institute of Field and Vegetable Crops, Maksima Gorkog 30, Novi Sad 21000, Serbia; E-Mail: sanja.vasiljevic@nsseme.com

**Keywords:** *Trifolium pratense* L., essential oil, antioxidant capacity, GC-MS analysis, *in vitro* experiments

## Abstract

The essential oils of three different growth stages of *Trifolium pratense* L. (TP1, TP2 and TP3) were investigated by gas chromatography-mass spectrometry and tested for their antioxidant and antimicrobial activities. The highest content of volatile compounds was found in the essential oil sample TP1, where terpenes such as β-myrcene (4.55%), *p*-cymene (3.59%), limonene (0.86%), tetrahydroionone (1.56%) were highlighted due to their biological activity. The antioxidant activity was determined by following the scavenging capacity of the essential oils for the free radicals DPPH^·^, NO^·^ and O_2_^·-^, as well as effects of the investigated oils on lipid peroxidation (LP). In all three cases, the sample TP1 showed the best radical-capturing capacity for DPPH^·^ (27.61 ± 0.12 µg/mL), NO^·^ (16.03 ± 0.11 µg/mL), O_2_^·−^ (16.62 ± 0.29 µg/mL) and also had the best lipid peroxidation effects in the Fe^2+^/ascorbate induction system (9.35 ± 0.11 µg/mL). Antimicrobial activity was evaluated against the following bacteria cultures: *Escherichia coli* (ATCC10526), *Salmonella typhimurium* (ATCC 14028), *Staphylococcus aureus* (ATCC 11632) and *Bacillus cereus* (ATCC 10876). None of the examined essential oil samples showed inhibitory effects on the tested bacterial strains.

## 1. Introduction

In recent years, the use of natural antioxidants found in plants has attracted interest due to their presumed nutritional and therapeutic value. Plants produce a vast and diverse assortment of organic compounds, the great majority of which do not appear to participate directly in their growth and development. These compounds are traditionally referred to as secondary metabolites. These plant natural products can be divided into three major groups: terpenoids, alkaloids, and phenylpropanoids and allied phenolic compounds [1]. Essential oils are volatile, natural, complex compound mixtures characterized by a strong odour and formed by aromatic plants as secondary metabolites. They are known for their antioxidant, antiseptic, bactericidal, viricidal, and fungicidal properties as well as their fragrance [2]. They are used in the preservation of foods and as antimicrobial, analgesic, sedative, anti-inflammatory, spasmolytic and locally anesthetic remedies. They can be synthesized by all plant organs and are stored in secretory cells, cavities, canals, epidermic cells or glandular trichomes. Several factors influence the chemical composition of plant essential oils, including the species, part of the plant, harvest season, geographical origin, the extraction method and others [3]. Plant volatile oils are generally isolated from plant material by different distillation methods and are mixtures of mainly terpenoids such as monoterpenes, diterpenes and sesquiterpenes and a variety of aliphatic hydrocarbons, acids, alcohols, aldehydes, acyclicesters or lactones [4]. In this work the chemical composition, antioxidant capacity, lipid peroxidation inhibition and antimicrobial activity of the essential oil obtained from *Trifolium pratense* L. was investigated.

*Trifolium pratense* L. (red clover) is the member of the family Leguminosae or Fabaceae. It is a short-lived biennial plant which has been used as food for livestock, but also as a health food for humans [5]. Interest in red clover has been reported since the early fifties. It was found that sheep in Australia that grazed red clover became infertile due to the great amounts of phytoestrogens it contained. The phytoestrogens present in red clover have a structure similar to that of endogenous 17β-estrogen and bind to the same receptors (ERα and ERβ). Therefore, this plant is used to treat and relieve symptoms that occur in postmenopausal women (hot flushes, cardiovascular health effects, breast cancers and osteoporosis). Many isoflavone preparations derived from red clover are available nowadays as nutritional supplements [6]. Red clover also has antioxidant activity that may be result of the presence of different flavonoids and other phenolic compounds such as phenolic acids, clovamides and saponins. It has also been used in traditional medicine to treat whooping cough, asthma, eczema and eye diseases [7].

There is relatively little available information about the composition of *T. pratense* essential oil and no literature reports about their antioxidant capacity. The essential oils of *T. pratense* obtained by steam distillation were analysed by Kami [8], who isolated about 80 compounds consisting of acids, phenols, aldehydes, ketones, alcohols, esters and hydrocarbons. Srinivas [9] identified 210 volatile constituents in CH_2_Cl_2_ extracts of *T. pratense*. In another study, 25 compounds from the leaves, flowers and seed of *T. pratense* were determined using GLC-MS analysis. Buttery *et al*. [10] described the major volatile components identified from the leaves (3-hexenyl acetate, 3-hexenol and β-ocimenes), flowers (acetophenone, methyl cinnamate and 1-phenylethanol) and from the seed pods (β-ocimenes, an unidentified sesquiterpene hydrocarbon and longifolene) Figueiredo *et al*. [11] investigated the volatile profile of three red clover forages (fresh plant, hay, silage) using GC and GC/MS analysis.

## 2. Results and Discussion

### 2.1. Chemical Composition of T. pratense Essential Oil

The chemical composition of the essential oil was analyzed using the GC-MS technique. Table 1 lists the chemical components of the investigated essential oils.

**Table 1:** Chemical composition of *Trifolium pratense* L. essential oil at three different stages of growth.

			Composition (%)			
No	Component	^a^ RI	TP1	TP2	TP3	^b^ Identification
1.	Hexane	604	1.70	-	-	RI, MS
2.	2-Pentanonone	680	-	6.66	-	RI, MS
3.	Methylbenzene	769	2.14	2.57	-	RI, MS
4.	1,3-Dimethylbenzene	867	1.10	-	-	RI, MS
5.	1,4-Dimethylbenzene	883	-	2.01	-	RI, MS
6.	Pentanoic acid	904	-	2.39	1.42	RI, MS
7.	7-Octen-4-ol	963	1.47	1.35	0.88	MS
8.	Beta-myrcene	990	4.5	-	-	RT, RI, MS
9.	Cyclopropane	n.d.	0.36	-	-	RI, MS
10.	Nonanal	1108	-	-	1.72	RI, MS
11.	2,4-Heptadienal	1011	-	-	0.35	RI, MS
12.	1-Bromocyclohexane	1023	-	-	1.28	RI, MS
13.	Fenchyl alcohol	1140	0.40	-	-	MS
14.	1,2,6-Hexanetriol	n.d.	-	-	0.56	MS
15.	*p*-Cymene	1026	3.59	-	-	RT, RI, MS
16.	L-Limonene	1030	3.86	-	-	RI, MS
17.	Benzaldehyde	1045	-	-	5.52	RI, MS
18.	Isobornyl thiocyanoacetate	1790	0.76	-	-	MS
19.	Decane	1005	0.39	0.44	-	RI, MS
20.	Undecane	1109	-	2.94	-	RI, MS
21.	Dihydrocarvone	1201	-	-	6.47	RI, MS
22.	Beta-ionone	1424	9.46	9.07	9.90	RT, RI, MS
23.	10-Methylnonadecane	1943	0.61	-	-	RI, MS
24.	2(4H)-Benzofuranone, 5,6,7,7a-tetrahydro-4,4,7a-trimethyl	1527	5.60	5.77	5.81	MS
25.	3-hexen-1-ol	1392	2.20	-	-	MS
26.	Megastigmatrienone	1560	-	-	16.10	RI, MS
27.	Hexadecane	1590	2.69	-	-	RI, MS
28.	Dodecanoic acid	1568	0.54	-	-	RI, MS
29.	2,6-Diisopropylnaphthalene	1728	7.51	5.44	1.76	RI, MS
30.	Tetradecane	1400	0.31	-	-	RI, MS
31.	Pentadecane	1498	-	-	0.91	RI, MS
32.	Isopropyl myristate	1830	-	0.51	-	MS
33.	Tetrahydroionone	1470	1.56	-	-	RI, MS
34.	Hexahydrofarnesyl acetone	1922	6.29	7.63	-	RI, MS
35.	Ocenol	2068	0.39	-	-	MS
36.	Phytol	2128	-	14.54	15.46	RI, MS
37.	4-Bromo-1-methyl-5-nitroimidazole	n.d.	0.46	-	-	RI, MS
38.	*n*-Hexadecanoic acid	1983	3.22	2.09	-	RI, MS
39.	Pentacosane	2493	-	3.81	-	RI, MS
	**Total identified**		**92.00**	**85.61**	**53.69**	
	Monoterpenes		12.00	-	6.47	
	Sesquiterpenes		29.90	30.86	23.91	
	Diterpenoids		25.56	24.53	9.90	
	Aliphatic compounds		13.34	20.20	4.85	
	Aromatic compounds		11.20	10.02	8.56	

^a^ Retention indices relative to C_9_–C_24_ *n*-alkanes on the HP 5MS column.; (n.d.) not detected; ^b^ (MS) mass spectrometry; (RT) comparison of the relative retention time with those obtained from the NIST/NBS, Wiley libraries spectra and literature data.

Thirty-nine different components were identified. The total number of chemical constituents identified in essential oils was 24 for *T. pratense* TP1, 15 for *T. pratense* TP2 and 14 for *T. pratense* TP3, representing 92.00%, 85.61% and 53.69% of the total oil contents, respectively. Based on the results shown in the Table 1 we can see that there is a wide range of known volatile compounds. Dominant compounds are aldehydes, alcohols and esters, which are the result of the action of fatty acid lipoxygenase, obtained within a few seconds after the occurrence of leaf and emitted immediately, as well as terpenes, which are synthesized *de novo* several hours or even days after damage [12,13]. The lipoxygenase pathway is described in other works [14]. Dudareva *et al*. [15] proposed that many volatile compounds are also formed by oxidation of the products of the initial transformation, dehydrogenation, alkylation, and other types of reactions in which among others, cytochrome P450 and NADP/NAD-dependent enzymes participate. These herbal volatile esters are synthesized by the effect of alcohol acyltransferases which catalyze the transfer of acyl groups from acyl-CoA intermediates to the hydroxyl group of alcohols. The table shows that the samples TP1, TP2 and TP3 contain common terpenes such as the diterpenoid β-ionone (9.46%, 9.07% and 9.90%, respectively) and the sesquiterpene 5,6,7,7-tetrahydro-4,4,7-trimethyl-2(4*H*)-benzofuranone (7.81%, 7.77% and 7.60%), where the sample TP1 shows the highest percentage of these compounds. The samples TP1 and TP2 present the acyclic sesquiterpene hexahydrofarnesyl acetone (6.29% and 7.63%, respectively), while TP3 does not present these compounds. The acyclic diterpenoid phytol (14.54% and 15.46%) is present only in samples TP2 and TP3. These differences can be explained by the plant being in a lower growth phase and thus containing a higher content of bioactive components. The monoterpenes β-myrcene (4.55%), *p*-cymene (3.59%), limonene (0.86%), and the diterpene tetrahydroionone (1.56%), were detected in a sample of TP1, while the oxidized monoterpene dihydrocarvone (2.47%) and the sesquiterpene megastigmatrienone (16.10%), were detected only in TP3. Differences in the contents of the three samples can be difficult to explain because of various biochemical processes (enzyme activity) that occur during the growth of the plants. Terpenes are not the most representative group of compounds present in the samples, but they are one of the most important classes of compounds that contribute to the antioxidant, antimicrobial, and many other pharmacological activities. The presence of the diterpene β-ionone (4.55%) and the monoterpene dihydrocarvone (6.47%) in *Trifolium pratense* L. was described by several authors [11], where β-ionone was present in a lower percentage than in our samples (1.49%) and megastigmatrienone, which shows cytotoxic activity, is also mentioned (0.89%) in this study. The presence of sesquiterpenes in *Trifolium pratense* L. was also reported in earlier work [12]. There is an opinion that the percentage of monoterpenes is reduced as plants age, as well as due to mechanical damage [16], which also coincides with our results, where the sample from the plant which is in a lower growth phase contains the highest percentage of β-ionone (4.55%). The samples TP1, TP2 and TP3 contained the following alcohols: 7-octen-4-ol (3.47%, 1.35% and 0.88%), 1,2,6-hexanetriol in TP3 (0.56%), which is mentioned in [10], and 3-hexen-1-ol (5.20%) which was present in the TP1 sample. It is believed that these alcohols come from the catabolism of fatty acids that accumulate in the plant. Benzaldehyde is the most abundant aldehyde and it is found in the TP3 sample (5.52%). It probably comes from the oxidation reactions of cinnamic acid or phenyl acetaldehyde [17]. Hexadecanoic acid is present in samples TP1 and TP2 (3.22% and 2.09%) and it is probably the result of enzymatic hydrolysis of esters [18]. These differences in the chemical composition between the samples are reflected in the fact that the essential oil content decreased with plant development, therefore essential oil obtained from the plant in lowest growth stage (TP1) presents the highest content of compounds.

### 2.2. *In Vitro* Antioxidant Activity of the Essential Oils

Reactive oxygen species (ROS) cause oxidative damage of cells and thus are involved in many human pathological diseases such as cancer, cardiovascular and neurodegerative processes, diabetes and others. One of the ways to scavenge and prevent the negative actions of ROS is the use of antioxidant molecules from Nature. The antioxidant activity of essential oils largely comes from presence of terpenes. They are among the most common natural products that show a wide range of biological and pharmaceutical activity. Terpenes have been shown to provide protection against oxidative stress and many diseases due of their antioxidant properties. The antioxidant properties of *T. pratense* essential oils were evaluated measuring their scavenging capacity toward DPPH, nitric oxide, superoxide anion radical, as well as their inhibition of lipid peroxidation in liposomes. The results are given in Table 2.

**Table 2:** IC_50_ values for evaluated antioxidant assays of examined *Trifolium pratense* essential oil, BHT and BHA.

	IC_50_ values for scavenging activity (µg/mL)			
Source	Radical species			LPx inhibition
Essential oil	DPPH^·^	NO^·^	O_2_^·-^	LPx
**TP1**	27.61 ± 0.12	16.03 ± 0.11	16.62 ± 0.23	9.35 ± 0.11
**TP2**	52.56 ± 0.28	25.31 ± 0.32	27.88 ± 0.34	15.27 ± 0.24
**TP3**	72.49 ± 0.14	41.69 ± 0.44	87.21 ± 0.12	36.81 ± 0.17
**BHT**	14.31 ± 0.32	8.46 ± 0.14	10.46 ± 0.13	26.15 ± 0.92
**BHA**	11.08 ± 0.28	6.31 ± 0.10	8.41 ± 0.12	36.08 ± 0.87

Values are means ± SD of five measurements.

Spectrophotometric determination of the neutralization of DPPH radicals is one of the most commonly used methods for rapid evaluation and preliminary assessment of the radical scavenging capacity of plant samples. All three of the investigated essential oil samples (TP1, TP2 and TP3) demonstrated the ability to reduce DPPH^•^ radicals. However, the best antioxidant activity of the essential oil was shown by TP1 (IC_50_ = 27.61 ± 0.12 µg/mL), which had an antioxidant capacity similar to that of synthetic antioxidants and better than that of the other two samples TP2 (IC_50_ = 52.56 ± 0.28 µg/mL) and TP3 (IC_50_ = 72.49 ± 0.14 µg/mL) which showed much lower antioxidant activity. Differences in the preliminary antioxidant activities of the tested essential oils depend on the plant growth stages, the method of plant material preparation or methods of obtaining the essential oils [19].

Superoxide anion radical occurs in the one-electron reduction of molecular oxygen or oxidation of the one-electron reduction product of hydrogen peroxide. A significant number of enzymatic reactions in biological systems, result in the formation of this radical species and its maximum amount is obtained in reactions involving oxidases (XOD, aldehyde oxidase) and in reactions catalyzed by NADPH-cytochrome C reductase, NADPH-cytochrome P450 reductase and others. In reactions between H_2_O_2_ and superoxide anion radical (Haber-Weiss or Fenton reaction) the OH^•^ radical is produced while the reaction with nitrogen (I)-oxide can form peroxynitrite anion (ONOO^−^), which may be more toxic then extracellular OH^•^ radicals [20]. From the obtained results of neutralization of O_2_^−•^ radicals and based on the calculated IC_50_ values it is evident that the synthetic antioxidants BHT (IC_50_ = 10.46 ± 0.13 µg/mL) and BHA (IC50 = 8.41 ± 0.12 µg/mL) used as reference compounds showed the best antioxidant capacity. Essentilal oil sample TP1 showed the best abiliy to inhibit O_2_^−•^ radicals (IC_50_ = 16.62 ± 0.23 µg/mL), but it was lower than that of the synthetic antioxidants BHT and BHA. Essential oil TP3 (IC_50_ = 87.21 µg/mL) showed significantly lower scavenging activity than the other two essential oil samples. Among the samples of the investigated oils of the tested plant species *Trifolium pratense* L. the best scavenging capacity to NO radical was displayed by the TP1 essential oil (IC_50_ = 16.03 ± 0.11 µg/mL) because its IC_50_ value was similar to the values of the synthetic antioxidants. The other two samples showed lower scavenging activity, especially sample TP3 (IC_50_ = 41.69 ± 0.44 µg/mL). Inhibition of nitric oxide radical (NO^•^) by essential oils of species of *Trifolium pratense* L. is very important as well as neutralization of superoxide anion radicals. In the reaction between O_2_^−•^ and NO^•^ peroxynitrite anion (ONOO^−^)is produced, which is very reactive, so in this respect, essential oil TP1 could be considered very suitable to neutralize superoxide anion and nitric oxide radicals. Since lipid peroxidation in the body is primarily the oxidative damage of cell membranes, as well as all other systems that contain lipids [21], in determining the overall antioxidant activity of different compounds, it is necessary (in addition to the antiradical assays) to examine their effect on the lipid peroxidation. The impact of various natural products (isolated compounds, extracts and essential oils) on lipid peroxidation can be studied in a number of different substrates (liposomes, linoleic acid, microsomes, various fatty oils, liver homogenate) [22]. Some substrates (liposomes and linoleic acid) are used more often than others primarily because of the simplicity of the method, but also because of easier dispersion in the investigated system compared to the fatty oil or microsome and hepatocytes isolation procedure. In this work liposomes were used. In the model of lipid peroxidation of liposomes, all tested essential oils showed the ability to inhibit lipid peroxidation. As in previous *in vitro* assays TP1 showed the most activity because it achieved a high IC_50_ value (IC_50_ = 9.35 ± 0.11 µg/mL) at a very low concentration. Sample TP2 was expressed also very great ability to inhibit LPx IC_50_ (IC_50_ = 15.27 ± 0.24 µg/mL). Both samples showed a better ability to inhibit LPx than the synthetic antioxidants BHT (IC_50_ = 26.15 ± 0.92 µg/mL) and BHA (IC_50_ = 36.08 ± 0.87 µg/mL). Unlike the others, in the LPx assay, the essential oil TP3 showed a good effect, with an IC_50_ value (IC_50_ = 36.80 ± 0.87 µg/mL) approximately the same as that of synthetic antioxidant BHA.

### 2.3. Antimicrobal Activity of T. pratense L. Essential Oil

The results of the antibacterial disc diffusion assays are summarized in Table 3. Essential oils possess antimicrobial activity due to their solubility in the phospholipid bilayer of cell membranes [23]. Although it is known that monoterpenes such as β-myrcene, *p*-cymene, limonene and dihydrocarvone [24] present in the investigated essential oils show antimicrobial activity, none of these samples were active against any of the tested bacterial strains. There is no report in the literature concerning the possible antimicrobial activity of essential oil obtained from *T. pratense*, but there are data on the antimicrobial activity of *T. pratense* L. extracts that did not show antibacterial and antifungal activity [25]. This result can probably be explained by the fact that investigated essential oils do not contain a sufficient amount of the terpenes that are responsible for antimicrobial effects.

**Table 3:** Antibacterial activity of *Trifolium pratense* L*.* essential oil (mg/mL).

	Inhibition zone diameter		
Bacterial strain	TP1	TP2	TP3
*Escherichia coli*	-	-	-
*Salmonella typhimurium*	-	-	-
*Staphylococcus aureus*	-	-	-
*Bacillus cereus*	-	-	-

## 3. Experimental

### 3.1. Chemical Reagents

Reagents such as thiobarbituric acid and (TBA), NADH, ethylenediaminetetraacetic acid (EDTA) and 2,2-diphenyl-1-picrylhydrazyl (DPPH), phenazine methosulfate (PMS) and trichloroacetic acid were purchased from Sigma-Aldrich Chem (Steinheim, Germany). *N*-(1-Naphthyl)-ethylenediamine dihydrochloride (NEDA) and ascorbic acid were acquired from Merck (Darmstadt, Germany). Sulfanilamide, 3,5-di-*tert*-butyl-4-hydroxytoluene (BHT), 3,5-di-*tert*-butyl-4-hydroxyanisole (BHA), were obtained from Fluka AG (Buchs, Switzerland), while L-ascorbic acid was from Merck. The commercial preparation of liposomes “PRO-LIPO S” was purchased from Lucas-Meyer (Hamburg, Germany). All chemicals used were of analytical grade.

### 3.2. Plant Material

The plant specimen material was collected in spring 2011. The collected plant material was dried in the shade and after drying, packed in paper bags in which it was kept until the experiments were performed. The plant material was *Trifolium pretense* L. at three different stage of growth: 30 cm (TP1), 50 cm (TP2) and the beginning of buttonization (TP3). The voucher specimen of the collected leaves (*Trifolium pratense* L. 1753 var. *sativa* Schreb. 1804, No 2-1751, No 2-1752, No 2-1753 (three growth stages), Novi Sad, Rimski Šančevi, UTM 34T DR 2 01, det.: Dr Goran Anačkov) were confirmed and deposited at the Herbarium of the Department of Biology and Ecology (BUNS Herbarium), Faculty of Natural Sciences, University of Novi Sad [26].

### 3.3. Microwave Hydrodistillation of Essential Oil

For the isolation of essential oils microwave-assisted hydrodistillation was used. This method has a few advantages over conventional extraction. First of all the extraction time is shorter. Using microwaves instead of steam, the interaction of electromagnetic fields with the liquid present in the walls of trichomes (glands which accommodate oil) leads to their cracking and rapid exit of the contents. This process is much slower in the case of classical hydrodestillation and the cells are pumped. Experiment were performed with a single-mode Discover BenchMate microwave reactor from CEM Corporation (Matthews, NC, USA) with a maximum output power of 300 W and with an IR temperature sensor positioned at the bottom of the cavity, below the vessel. The microwave reactor covers a variety of reaction conditions in open- (up to 125 mL) and closed-vessel systems (up to 50 mL filling volume). Reactions are performed in the open-vessel system by using a Clevenger condenser instead of the standard Liebig condenser with the aim of collecting final products/oils and this was only modification that we made to ensure the safety of performing these experiments. Since the experiments were carried out in temperature control mode, the microwave magnetron power constantly regulates itself, from a maximum value at the beginning of heating (thus reaching the desired temperature faster) to a lower one after the set temperature of 90 °C is reached and also during the experiment to keep the sample of water heated at 90 °C. Previously chopped and dried plant material (4 g) was placed in the flask (100 mL) and coated with 50 mL of distilled water. Hydrodistillation lasted 15 min, and oil was collected in *n*-hexane. Essential oil solution in *n*-hexane was dried with sodium sulfate and the *n*-hexane removed on a rotatory vacuum evaporator. The obtained essential oil was kept in sealed bottles in the fridge (+4 °C) for one week. Before experiments, the oil was dissolved in *n*-hexane to make up series of solutions (w/v) for the antioxidant assays and solution for GC-MS analysis.

### 3.4. GC-MS Analysis of T. pratense Essential Oil

Qualitative analysis was performed using an Agilent 6890 N gas chromatograph (GC) equipped with Agilent 5973 mass selective detector (MSD), Agilent Autosampler 7683 and Agilent DB-5MS capillary column (30 m, 0.25 i.d., 0.25 µm film thickness) (Agilent Technologies, Santa Clara, CA, USA). The MS detector was operated in electron impact (EI) mode at 70 eV with interface temperature of 280 °C; the scan range was 50–550 amu. The injection port temperature was set at 250 °C. GC was performed in splitless mode; carrier gas was helium at a constant flow rate of 1 mL/min. The column temperature was programmed as follows: an initial temperature of 60 °C increased to 280 °C at rate of 3 °C/min. The injection volume was 1.0 µL. The identification of individual compounds was based on comparison of their mass spectra with those obtained from the NIST/NBS, Wiley Libraries spectra, and confirmed by comparison of Kovats retention indices (KI) with literature data [27]. Diesel oil (a mixture of C_8_-C_28_ *n*-alkanes corresponding to 800–2,800 KI) used as a standard for determination of retention indices.

### 3.5. *In Vitro* Antioxidant Activity Assays

#### 3.5.1. The DPPH Assay

The DPPH assay was performed as described before [28], following the transformation of stable violet DPPH radical in its yellow reduced DPPH-H neutral form (DPPH-H). The samples (from 2.50 to 125 μg/mL) were mixed with 90 μM DPPH^•^ solution (1 mL) and filled up with 95% MeOH to a final volume of 4 mL. The mixture was left to stand at room temperature for 1 h and afterwards the absorbance of the resulting solutions was read spectrophotometrically at 515 nm against the blank probe that contains all mentioned chemicals except the sample. The synthetic antioxidants butylated hydroxytoluene (BHT) and butylated hydroxyanisole (BHA) were used as positive controls.

#### 3.5.2. Neutralization of Super Oxide Anion Radical

Measurement of superoxide anion scavenging activity of *Trifolium pratense* L. essential oil was based on the method described by Cos *et al*. [29]. Superoxide radicals are generated in PMS-NADH systems by oxidation of NADH and assayed by the reduction of nitroblue tetrazolium (NBT). The reaction mixture containing NBT (0.2 mL, 144 µmol/L), essential oil (10 µL, concentrations ranging from 2.50 to 125.0 µg/mL, substituted with solvent in the control experiments), NADH (0.1 mL, 0.68 mmol/L), and freshly prepared PMS (60 µmol/L) in phosphate buffer (1.1 mL, pH 8.3). Blank probe was prepared by mixing buffer (1.5 mL) and extract (10 µL). Absorbance was measured at 560 nm after 5 min.

#### 3.5.3. Nitric Oxide Scavenging Activity

This assay was based on the method of Green *et al*. [30], adapted for 96-well microplates. Briefly, the reaction mixture containing sodium nitroprusside (10 mmol/L, 75 µL), phosphate buffer, pH 7.4 (75 µL) and extract (10 µL, concentration ranging from 2.50 to 125.0 µg/mL) or standard solution (BHT or BHA) was incubated at 25 °C for 90 min. Essential oil (10 µL) and buffer (150 µL) were used in the blank probe. After incubation, solution prepared by mixing equal amounts of Griess reagent (2% sulfanilamide 4% phosphoric acid and 0.2% *N*-(1-naphthyl) ethylenediamine dihydrochloride, 150 µL) was added to the reaction mixture and allowed to stand for 3 min. The absorbance of chromophore (purple azo dye) formed during reaction between nitrite ions sulphanilamide and subsequent coupling with *N*-(1-naphthyl)-ethylenediamine dihydrochloride (NEDA) with was measured at 546 nm against the blanks. The percentage of RSC for each radical was calculated using the following equation:

RSC (%) = 100 × (A _blank_ − A _sample_/A _blank_) (1)

From the obtained RSC values, the IC_50_ values, which represented the concentrations of the examined extracts that caused 50% neutralization, were determined by linear regression analysis.

#### 3.5.4. Lipid Peroxidation Assay

Malondialdehyde (MDA) is one of the final products of lipid peroxidation (oxidative damage of membrane lipids). Therefore, the extent of LP was determined by measuring the color intensity of the adduct produced in the reaction between MDA and 2-thiobarbituric acid (TBA) by the TBA assay [31]. The commercial preparation of liposomes “PRO-LIPO S” (Lucas-Meyer) pH = 5–7 was used as a model system of biological membranes. The liposomes, 225–250 nm in diameter, were obtained by dissolving the commercial preparation in demineralized water (1:10) in an ultrasonic bath. Five concentrations of essential oils (dissolved with *n*-hexane) were prepared for the experiment. The content of the MDA (TBARS) was determined by measuring the absorbance of the adduct at 532 nm. In a test tube, a suspension of liposomes (50 μL) was incubated with 0.01 M FeSO_4_ (20 μL), 0.01 M ascorbic acid (20 μL), and essential oil samples (10 μL) in 0.05 M KH_2_PO_4_-K_2_HPO_4_ buffer (2.90 mL, pH 7.4, 3 mL final solution). Samples were incubated at 37 °C for 1 h. LP was terminated using the reaction with TBA reagent (1.5 mL) and EDTA (0.2 or 0.1 mL), heated at 100 °C for 20 min. After precipitated proteins were cooled and centrifuged (4,000 rpm for 10 min), the content of the MDA (TBARS) was determined by measuring the absorbance of adduct at 532 nm. Analyses were compared with the commercial synthetic antioxidant BHT (0.1 M stock solution, concentration 44.0 μg/mL) as a positive control. Five replicates were performed for each sample. The control with *n*-hexane was also analyzed. The percentage of LP inhibition was calculated by the following equation:

*I* (%) = (*A*_0_ − *A*_1_)/*A*_0_ × 100 (2)

where *A*_0_ is the absorbance of the control probe (full reaction, without the test compound) and *A*_1_ is the absorbance in the presence of the inhibitor.

### 3.6. Determination of Antimicrobial Activity of T. pratense Essential Oil

The *in vitro* antibacterial activities of the essential oil and its major compounds were evaluated against the following bacterial cultures: *Escherichia coli* (ATCC10526), *Salmonella typhimurium* (ATCC 14028), *Staphylococcus aureus* (ATCC 11632), and *Bacillus cereus* (ATCC 10876). The cultures of the test bacteria were grown 20–24 h in Müller-Hinton agar (Torlak, Belgrade, Serbia) at 37 °C. Bacteria were obtained from the stock cultures of Microbiology Laboratory, Faculty of Technology, University of Novi Sad. The agar disc diffusion method was used for the evaluation of the antibacterial activity of the samples. The strains were grown on Mueller-Hinton agar slants at 37 °C for 24 h and checked for purity. After the incubation, the cells were washed off the surface of agar and suspended in sterile physiological solution. The number of cells in 1 mL of suspension for inoculation measured by McFarland nephelometer was 5 × 10^7^ CFU/mL. One mL of these suspensions was homogenised with melted (45 °C) Mueller-Hinton agar (9 mL) and poured into Petri dishes. On the surface of the agar, 6 mm diameter paper discs (HiMedia^®^, Mumbai, India) were applied and impregnated with 15 μL of essential oil (concentration 100 µg/mL). The plates were incubated 48 h at 37 °C and the diameters of the resulting zones of inhibition (ZI) were measured and expressed in mm. The evaluation of the antimicrobial activities of the samples was carried out in triplicates.

## 4. Conclusions

Examining the content of essential oil by GC-MS analysis, it can be concluded that the samples contain various volatile compounds and terpenoids which contribute to their biological and pharmacological activities. TP1 contains the highest percentage of the monoterpenes β-myrcene (4.55%), *p*-cymene (3.59%), and limonene (0.86%), while the oils TP2 and TP3 contain smaller amounts of sesquiterpenes which contribute less to their antioxidant activity. Comparing the IC_50_ values obtained in determining the antiradical capacity of the essential oils tested on several forms of radicals a striking common feature is that the essential oil TP1 shows significantly better scavenging activity than the other two samples of essential oils. These results are probably caused by the fact that the vegetative development of the plant affects the chemical composition of the essential oil of plants. In our case, this means that the essential oil isolated from the plant which is in its lowest level of development, showing the best antioxidant activity, probably due to the presence of a higher content of bioactive molecules. During a plant growth these substances probably undergo many biotransformations that lead to many changes in the content of chemical components, which are difficult to explain.
